# Sudden death in a young female with an under-recognised coronary anomaly

**DOI:** 10.1186/1746-1596-8-41

**Published:** 2013-02-28

**Authors:** Fabio De-Giorgio, Vincenzo M Grassi, Giuseppe Vetrugno, Vincenzo Arena

**Affiliations:** 1 Institute of Legal Medicine, Catholic University, Medical School, Rome, Italy; 2 Institute Pathologic Anatomy, Catholic University, Medical School, Rome, Italy

**Keywords:** Sudden death, Agenesia, Septal coronary branches, Heart, Left descending artery

## Abstract

Anomalies of coronary number and course represent an opinion-dividing topic in cardiopathology, particularly for their relationship with sudden cardiac death.

To the best of our knowledge, we herein report the first fatal case of a young female whose coronary anatomy was characterised by the absence of any septal perforator branch in the proximal segment of the LAD.

This case could be useful for pathologists, coronary angiographers, and interventional cardiologists in detecting this infrequent anomaly, thus providing a more accurate estimation of its incidence.

**Virtual Slides:**

The virtual slide(s) for this article can be found here: http://www.diagnosticpathology.diagnomx.eu/vs/3570015858473043

## Introduction

Anomalies of the coronary arteries represent some of the most opinion-dividing topics in cardiopathology, particularly due to their relationship with sudden cardiac death, which frequently remains unclear.

**Figure 1:**
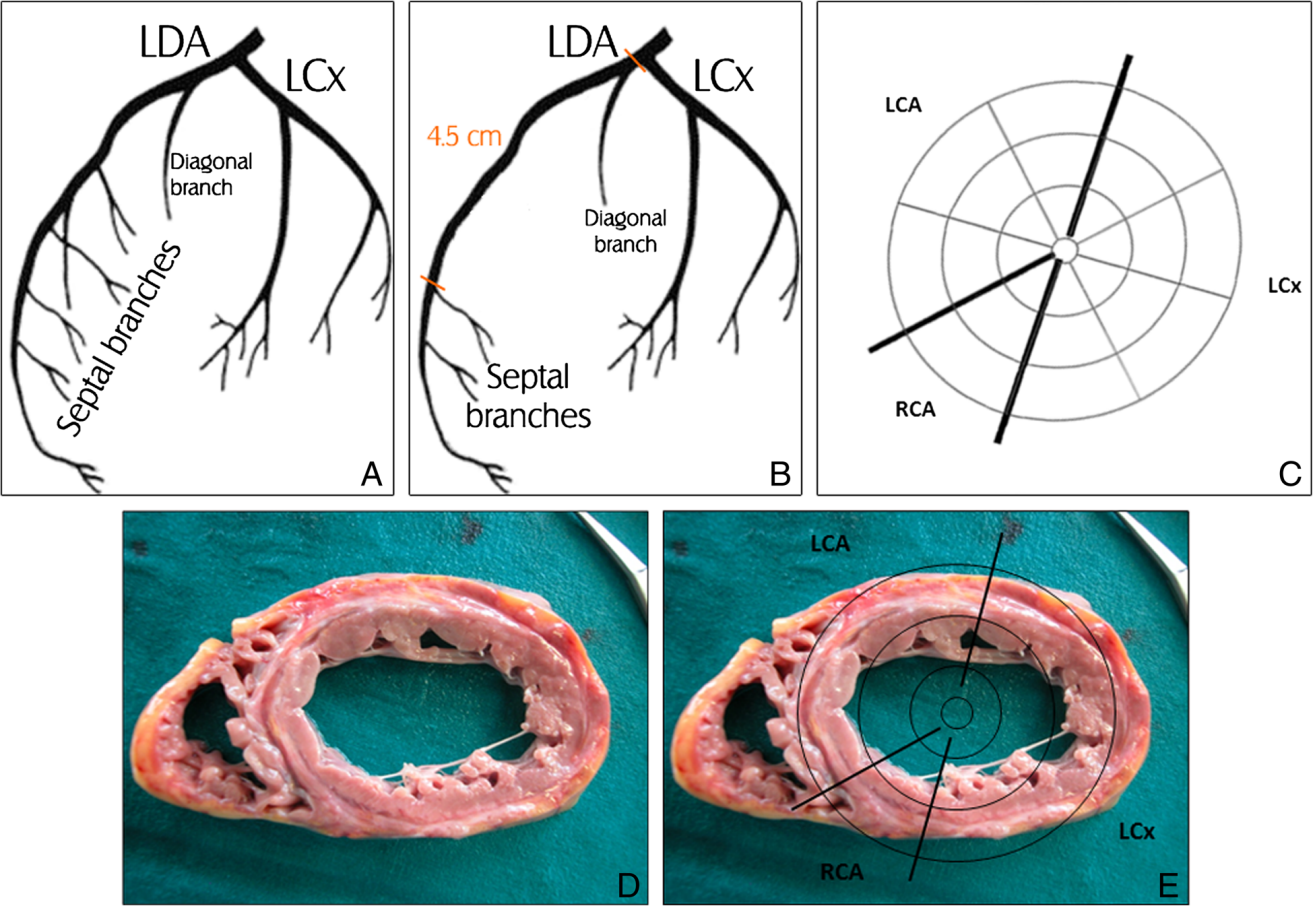
**1A: Drawing of the lateral view of the normal left coronary artery.** 1**B**: Drawing of the lateral view of the left coronary artery as observed at autopsy. 1**C**: Schematic illustration of the left ventricle showing the three main coronary arteries and their tributary areas. 1**D**: Transverse section of the heart showing a wide infarcted area of the anterior portion of the interventricular septum. 1**E**: Superimposition of 1**C** and 1**D**. LCA= Left Coronary Artery. LDA= Left Descending Artery. LCx= Circumflex Artery. RCA= Right Coronary Artery.

An anomaly of the coronary arterial anatomy can be defined as any coronary pattern with a feature rarely encountered in the general population [1]. Normally, the left anterior descending artery (LAD) lays in the anterior interventricular groove and provides the anterior septal perforator branches [2], while either the right coronary artery (RCA) or the circumflex artery (LCx) provides the posterior septal perforator branches through the posterior descending branch [3]. Thus, the LAD is responsible for most of the blood flow to the interventricular septum, the RCA for the atrioventricular node and the posterior descending artery for the posterior portion of the interventricular septum [4]. The first septal artery typically arises from the proximal segment of the LAD (Figure 1) and is considered by angiographers the most important landmark along the course of the LAD to separate the first segment of the LAD from its middle segment [5]. Septal vessels have a horizontal or oblique course extending throughout the epicardium and the myocardium to the endocardium; this course is approximately perpendicular to the cardiac wall and parallel to the anteroposterior (transverse) plane of the interventricular septum. A complex anastomotic system connects the homocoronary and intercoronary collaterals [3,4].

Septal branches can be classified into two categories according to their structure: straight vessels or branching vessels. Straight vessels cross the whole myocardial wall, while branching vessels have branches at different wall levels [2].

We herein report the case of a young female whose coronary anatomy was characterised by the absence of any septal perforator branch in the proximal segment of the LAD.

### Case history

A previously healthy 16-year-old Caucasian woman suddenly collapsed during exercise. She was taken to the Emergency Department, but she was declared dead on arrival. In the pathological anamnesis, collected from her parents, no hereditary history of sudden death was reported. The young woman did not smoke, drink, or use drugs. The parents only reported that she had already collapsed during exercise one year earlier but suddenly became conscious. No medical examinations were performed because the collapse was evaluated as a transient hypotensive or hypoglicaemic event. Otherwise, the girl never complained of any clinical symptoms.

An external examination demonstrated that the body constitution was normal according to the age. At autopsy, all organs were unremarkable, except the cardiovascular system. The heart weighed 280 g (normal values in 16-year-old women: 200–258 g [6]) and measured 15 cm in length and 12 cm in width. There were no signs of pericarditis. The thickness of the left ventricle wall was 14 mm (normal value in women: 15 mm [6]), while the right ventricle wall measured 3 mm in thickness (normal value in women: 2–4 mm [6]); the cut surface showed white macroscopically evident areas of scarring replacing the myocardium as well as pale and dry areas. The anterior portion of the interventricular septum presented a wide infarcted area (Figure 1).

**Figure 2:**
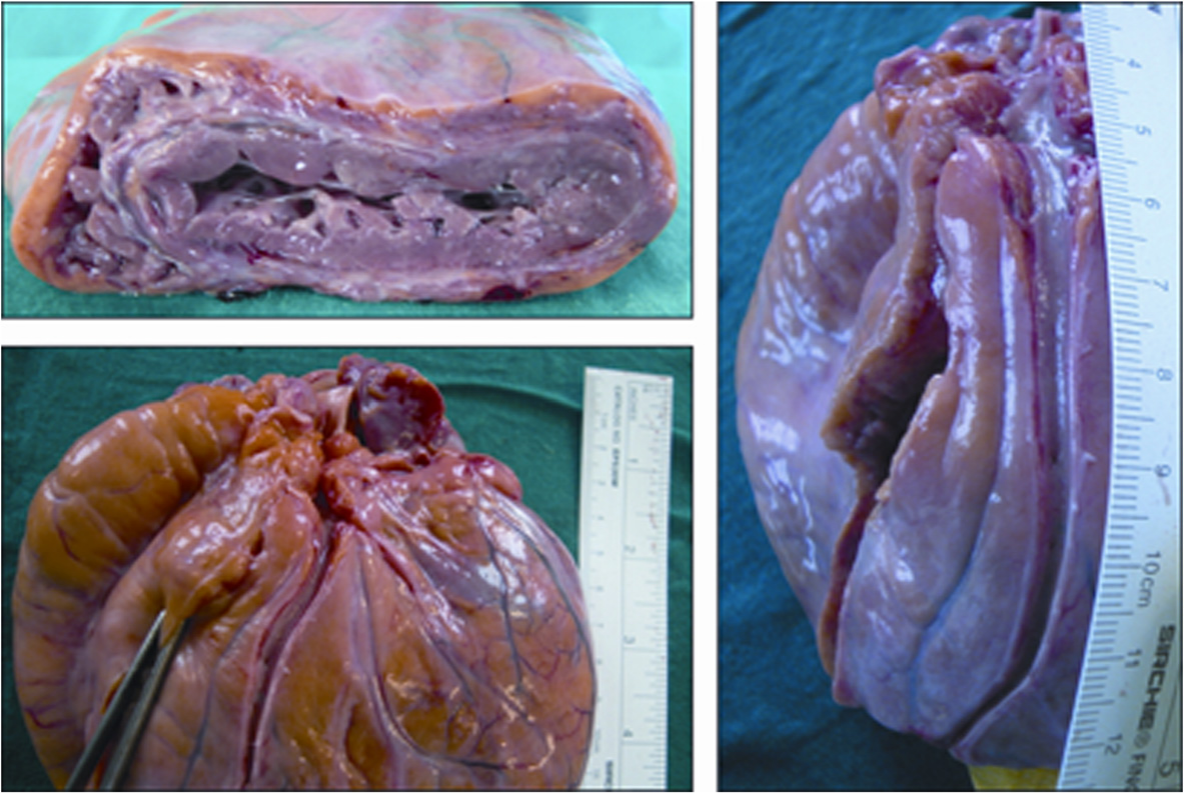
**Left ventricular wall and LAD.** The vessel did not reveal any septal perforator branches for 4.5 cm from its origin at 1 cm from the proximal common trunk.

The heart valves were normal; the coronary arteries were regular in their origin and size and no thrombi were found within their lumen. Notably, the LAD did not reveal any septal perforator branch in its proximal segment. In fact, the left coronary artery ran for 1 cm and then bifurcated in the LAD and the LCx; the LAD had a septal branch 4.5 cm from its origin (Figures 1 and 2).

**Figure 3:**
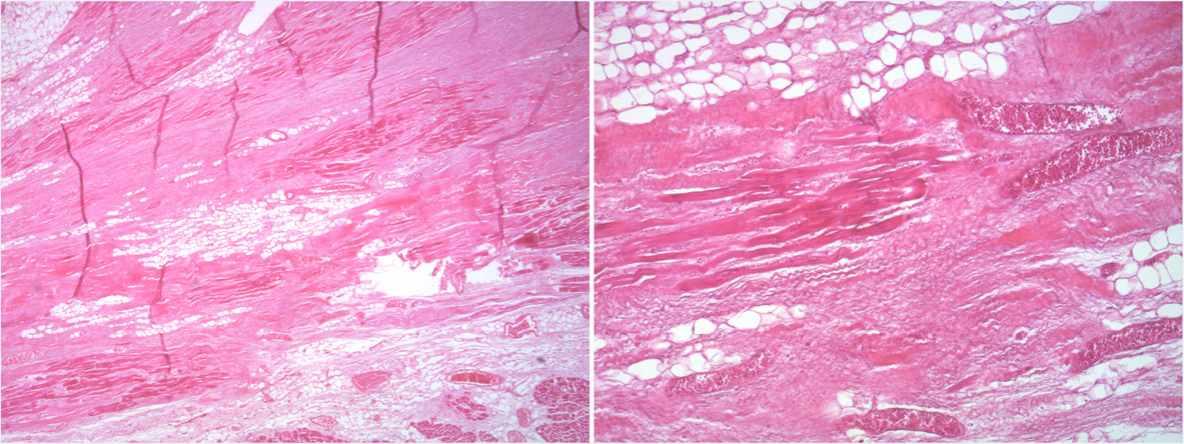
Microscopic evaluation showing myocardiosclerosis and fibrofatty replacement associated with waving of the myocytes.

A histological evaluation showed areas of diffuse regressive alterations, characterised by the presence of myocardiosclerosis and fibrofatty replacement, associated with waving of the myocytes as per acute ischaemic damage (Figure 3).

Toxicological screening for common drugs of abuse (heroin, cocaine, alcohol, cannabinoids, and benzodiazepines) was negative. Based on the autopsy findings and the histological and toxicological results, the ultimate cause of death was myocardial ischaemia, while the underlying cause was identified in the coronary anomaly characterised by the absence of any anterior septal perforator branch in the proximal segment of the LAD.

## Discussion

The most common causes of sudden cardiac death can be grouped into five categories: coronary disease, myocardial pathology, functional disease, pathology of the great vessels and miscellaneous, i.e., myxoma, cardiac metastases, or pericardial effusion/tamponade [7,8]. Congenital anomalies of coronary artery number and course are part of the first category and can be classified on the basis of their origin, course, size, and termination [1,9]. According to the guidelines published by the Association for European Cardiovascular Pathology, a diagnosis of sudden cardiac arrest is supported only by a post-mortem finding of coronary artery occlusion [10]. Therefore, in the presence of non-atherosclerotic acquired coronary artery diseases, a definitive diagnosis of sudden death can be formulated only after a post-mortem finding of coronary dissection or vasculitis complicated by occlusive thrombosis [11]. Congenital anomalies of the coronary arterial anatomy could however be identified as the real cause of sudden death when the tributary myocardium exhibits an acute infarct or post-infarction scar [11].

Notably, in the case we presented, there was a correspondence between the myocardial area affected by acute ischaemic damage (i.e., the anterior interventricular septum) and the absence of septal branches in the proximal segment of the LAD. Apart from this anomaly, the coronary arteries were perfectly normal in origin, course, size, and lumen patency.

Angelini et al. [1] included the ectopic origination of the first septal branch in the classification of coronary anomalies observed in normal human hearts. Dabizzi et al. [12] reported the absence of the first septal branch in a patient suffering from tetralogy of Fallot with a single coronary ostium or LAD arising from the RCA; in these cases, angiography showed that the interventricular septum had a blood supply similar to other individuals.

To our knowledge, no cases of sudden death in the presence of the anomaly presented in this publication have been reported. As demonstrated by Dabizzi et al. [12], in our case, the septum could have been supplied by an aberrant arising septal branch. Whatever the case may be, this vessel was not macroscopically identified. However, the amount of blood delivered was so small that it was impossible to satisfy the tissues’ blood request. In fact, histology identified signs of both chronic and acute myocardial ischaemic damage.

Arrhythmogenic right ventricular cardiomyopathy was excluded according to the absence of myocyte loss, fibrofatty replacement, and chronic inflammatory infiltrates [13].

The clinical history, autopsy, and histological results suggest arrhythmic sudden cardiac death triggered by myocardial ischaemia due to the absence of any anterior septal perforator branch in the proximal segment of the LAD.

In conclusion, we believe that this case is of potential interest for pathologists and coronary angiographers and interventional cardiologists. In fact, the agenesis or the ectopic origin of the anterior septal branches can be considered as a potential source of sudden death or cause of death. Moreover, when no other significant autopsy findings are present, particularly in the absence of risk factors and pathological findings directly related to the death, this anatomical variant should be considered. Finally, this case could be useful to autopsy pathologists in detecting this infrequent anomaly, thus providing a more accurate estimation of its incidence.

## Ethical approval

The authors declare that the present case-report has been carried out in compliance with the Helsinki Declaration (http://www.wma.net/en/30publications/10policies/b3/).

## Competing interests

The authors declare that they have no competing interests.

## Authors’ contributions

All authors equally contributed to the bibliographic research and to the writing of the manuscript. All authors read and approved the final manuscript.
